# Promoting Rural Tourism in Inner Mongolia: Attributes, Satisfaction, and Behaviors among Sustainable Tourists

**DOI:** 10.3390/ijerph18073788

**Published:** 2021-04-05

**Authors:** Chen Che, Bonhak Koo, Jiatong Wang, Antonio Ariza-Montes, Alejandro Vega-Muñoz, Heesup Han

**Affiliations:** 1 College of Hospitality and Tourism Management, Sejong University, 98 Gunja-Dong, Gwanjin-Gu, Seoul 143-747, Korea; cecencan@naver.com; 2 School of Hospitality and Tourism Management, Spears School of Business, Oklahoma State University, 365 Human Sciences, Stillwater, OK 74078, USA; bkoo@okstate.edu; 3 College of International Business, Zhejiang Yuexiu University, No.428 Kuaiji Road, Yuecheng District, Shaoxing 312000, China; wjt521andy@hotmail.com; 4 Social Matters Research Group, Universidad Loyola Andalucía C/Escritor Castilla Aguayo, 4, 14004 Córdoba, Spain; ariza@uloyola.es; 5 Public Policy Observatory, Universidad Autónoma de Chile, Santiago 7500912, Chile; alejandro.vega@uautonoma.cl

**Keywords:** destination attributes, tourist satisfaction, revisit intention, rural tourism, Inner Mongolia

## Abstract

With the growth of rural tourism in China, this study aims to determine the destination attributes, tourism satisfaction, and intention of revisiting Inner Mongolia. This study also investigated the mean comparison of tourist satisfaction and revisit intention across domestic tourists’ demographic characteristics. Structural analysis revealed that destination attributes have a positive influence on satisfaction and revisit intention. In addition, the result of the mean difference test showed that satisfaction is significantly different between male and female tourists, and revisit intention significantly varies across the season. Our findings have an excellent directive significance to bring forward rural tourism in Inner Mongolia.

## 1. Introduction

In recent decades, a primary current focus in increasing rural destination economic development is to ensure rural tourism sustainability [1,2,3,4]. With the growth of rural tourism in China, it has become a popular form of travel among visitors, which significantly contributes to revenue in tourism [3]. In 2019, the Ministry of Culture and Tourism (MCT) in China had started to promote rural tourism and released 320 key villages where rural tourism was being provided. The following year, the MCT added 680 key villages as well. In the list of key villages in rural tourism, there are 24 villages in Inner Mongolia [5,6]. As an essential hub of “One Belt And One Road” construction, which is a Chinese government’s global infrastructure development project, Inner Mongolia’s rural tourism has started to provide a unique experience to the world in various ways.

Rural tourism in China has become an increasingly important industry to fight against poverty in rural areas, and therefore it helps Chinese villages embrace prosperity [1,7]. In this regard, rural tourism destination has attracted a great number of both domestic and international tourists [3]. Previous studies have shown the importance of destination attributes on tourist behavior intention [8,9]. According to Lu [2], ancient villages have increasingly attracted tourists and have become a thriving cultural tourist destination. Therefore, the list of key villages representing a new type of rural tourism will play an essential role in promoting the revitalization of rural areas. Compared to the modern lifestyles and familiar tourists’ choices, the remote regions’ heritage, local culture, and myths constitute an engaging and memorable tourism experience [10,11]. According to Eom et al. [12], the local foods and restaurants, natural environment and landscape, and activity and events help destinations in rural areas have a competitive advantage. In particular, after the COVID-19 outbreak was brought under control in the country and various sectors have started resumption, an increasing number of Chinese travelers have opted for rural destinations to get closer to nature and experience a slow-paced lifestyle instead of urban sightseeing tours [10,13]. 

Despite the emerging significance of the destination attribute with its sustainable advantages, little research has examined the impact of weather and climate, place attachment, and tourist satisfaction in explicating revisit behavior. Furthermore, there is scant research on rural destination attribute and tourist behaviors in Inner Mongolia. Although there are abundant tourism resources in Inner Mongolia, examining Inner Mongolia tourism in tourism development is still a blind spot. 

To fill the research gap, this study aims to (1) examine the effect of destination attribution (place attachment, physiography climate, minority culture, infrastructure, service, culture events) and tourist’s satisfaction; (2) investigate the relationship on destination attribution (place attachment, physiography climate, minority culture, infrastructure, service, culture events) and tourists’ revisit intention to Inner Mongolia; and (3) explore the tourists’ satisfaction and their revisit intention this destination. This study contributes to understanding tourists’ intention to revisit Inner Mongolia by understanding destination attribution and satisfaction with travel. Finally, this study speculates the attractions driving the development of rural tourism in Inner Mongolia.

## 2. Literature Review

### 2.1. Rural Tourism in Inner Mongolia

Rural tourism has become an essential engine for development in rural regions that expands the concept of tourism [3,4,14]. Tourists are inspired to visit the rural regions through a wide range of desires that include the potential of having fun, soothing, and restoring. In particular, travel to a rural region provides a localized experience: experiences of nature, environments, residents, traditional cuisine, local specialties, authentic customs, slow speed, intimacy, tranquility, and reconnection to self [15]. Definitions of rural tourism vary from region to region [14]. China is one such nation that has pursued rural socio-economic regeneration by encouraging rural tourism, as the Chinese government is motivated by socio-economic needs to produce revenue to resolve China’s poverty problems [3]. Recently, rural tourism has become the top option for Chinese people for recreation and leisure, with both cost and protection considered in the sense of the life-threatening COVID-19 pandemic [13]. 

**Figure 1:**
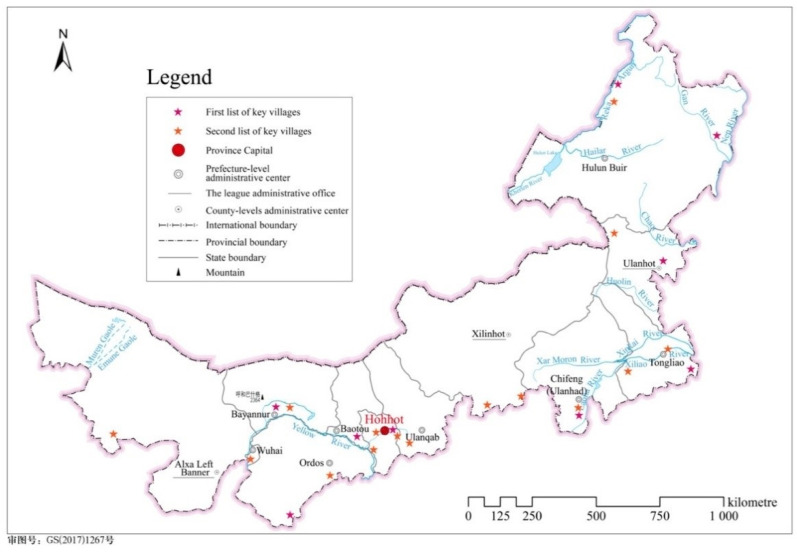
Rural tourism key villages in Inner Mongolia in 2020. Note: This drawing is based on the standard map obtained from the standard map service system of the Ministry of Natural Resources of the People’s Republic of China (drawing review No.: GS (2017) No.1267). The base map has not been modified.

Thus, rural tourism has been increasing as a new form of tourism in China. Table 1 shows that there has been a gradual increase in the number of Inner Mongolia tourist reception and income of tourism since 2010. In particular, domestic tourism is the primary income source. Based on the development plan for tourism in the country’s 13th five-year plan and a guideline of the State Council on the vitalization of rural industries, China’s Ministry of Culture and Tourism (MCT) published key villages in 2019 and 2020. According to the ministry, the key villages were chosen based on unique yet competitive places that possess rich cultural and tourism resources, boast sound protection and conversion of traditional rural culture, and have a mature and high-quality tourism product system. The key villages also should guarantee a beautiful ecological environment with well-rounded infrastructure and public services. Inner Mongolia has 24 villages out of 1000 villages in China (Figure 1).

### 2.2. Destination Attributes and Satisfaction

The destination attribute is defined as an amalgam of the different elements of a tourism destination that attract tourists to the destination [16,17]. Destinations are composed of various attributes, and tourists can experience different destination attributes accordingly [18]. Investigating destination attributes is essential for visitors to select a destination, and tourists can compare destination attributes to choose the best travel destination [12,19]. As such, a destination’s unique characteristics include various attributes, including its landscape and activities [9]. Multiple destination characteristics are also important antecedents of visitors’ satisfaction and revisit intentions [9,20,21]. As mentioned above, destination attributes affect the destination’s image formation [8]. Tourists collect more information to compare destinations’ attributes to have more options to maximize the benefits for them, including different gastronomic traditions, geography environment, region, customs, or natural conditions [22,23]. Those multiple destination attributes were dominant to determine the tourist emotional arousal, tourism experience, and memorability [24]. Previous studies have shown that multiple destination attributes are vital for tourist satisfaction and return [25,26,27]. 

The satisfaction of tourism is defined as the overall acceptance of experience and product or service by customers affecting their purchase decision [28]. Satisfaction is considered an important variable due to its high effects on customers’ future behavior and attitudes about specific products or services [29]. Many studies in different disciplines have investigated and tested the relationships between the overall risk construct and customer satisfaction [30,31]. In particular, researchers have studied the association between destination attributes and tourist satisfaction [25,32]. Most of the research has proved that they have a significant relationship. Cervi [33] highlighted the importance of social networks and tourist emotions of destination. The tourist satisfaction with the destination attributes is strongly influenced by intent to recommend the destination to other people and intention to return [25]. Tourists who stay at the destination experience various products, local events, or services and may evaluate each aspect separately [34,35,36]. In this regard, the following hypothesis is presented:

**Hypothesis** **1.** *Destination attributes of Inner Mongolia have a significant and positive influence on tourists’ satisfaction.*

### 2.3. Destination Attributes and Intention to Revisit

Destination attractiveness is commonly regarded as one of the critical determinants of destinations’ competitiveness [17,37]. Crouch [38] demonstrated that the core tourism resource and destination attributes are the foundation of destination competitiveness. Moon and Han [9] found that island destination attributes can stimulate tourist satisfaction and revisit intention. In a recent study, Eom et al. [12] pointed out that the dimensions of multiple attributes play important roles in engaging travelers to visit the destination.

The intention of revisit can be described as tourists’ desire or intentions to revisit the same place [39]. In addition, traveler’s revisit intention is a significant predictor because the expense of keeping tourists is significantly cheaper than the expense of attracting new tourists [40,41]. Current research has confirmed the relationships between destination attributes and the intent to revisit. Meng and Han [42] investigated working-holiday tourist behaviors. The basis of their study described a significant relationship between working-holiday travelers and behavior intention. Existing studies supported the relationships among destination attributes and revisit intention [9,12]. Centered on the results as mentioned above, the following hypothesis is developed:

**Hypothesis** **2.** *Destination attributes of Inner Mongolia have a significant and positive influence on tourists’ revisit intention to Inner Mongolia.*

### 2.4. Demographics, Satisfaction, and Intention to Revisit

The previous literature’s demographic concepts are related to the population and distribution of primary attributes, such as gender, age, and education [43,44,45]. In tourism research, researchers frequently collect information in terms of the length of trip, past trip experience, travel companion, type of transportation, or the purpose of the visit destination to identify tourists’ behavioral characteristics [46,47]. Participants’ demographics play an important role in tourist behaviors. Tsiotsou and Vasioti [48] investigated the relationship between demographic and leisure activities; the basis of this research was that education and age discriminate consumers’ satisfaction with tourism service. Ozdemir et al. [49] verified that tourist profile and tourism behavior variables influenced tourist satisfaction. 

Gender analysis provides information on destination marketing, destination tourism, travel experience, and differences between females and males. For example, Wang and Hsu [50] found that gender influences travel motivation, advertising, and word-of-mouth on the cognitive and affective image. Pung et al. [51] studied the transformative travel experience for both females and males using a double duo-ethnography approach to analyze young males’ and females’ travel experience of transformation. The previous studies verified and validated gender differences in tourist behavior literature [49,52]. 

**Figure 2:**
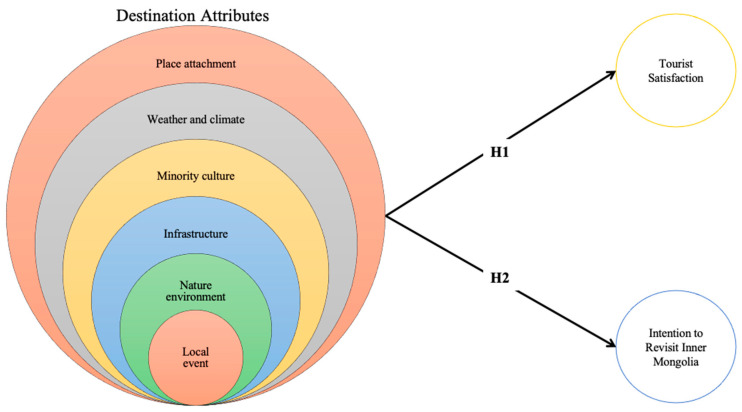
Proposed model.

Tourists can be divided not only by gender but also by age. Several researchers have studied the customer’s behavior intention by different age groups to determine age differences [53,54,55]. To this end, Figure 2 displays the proposed model, including all the hypotheses in this research.

## 3. Methodology

### 3.1. Measurements Instruments

The measure items were adopted from existing studies [8,9,52,56,57,58,59]. A seven-point Likert scale was used for the survey questionnaire that ranged from (1) “strongly disagree” to (7) “strongly agree.” A seven-point Likert scale is highly recommended for tourism quality-related items due to its functionality in providing accurate outcomes [60]. The questionnaires consist of three parts. The first part describes rural tourism in Inner Mongolia to help respondents to understand it. The second part includes the measurements of the major scale items. The last part is about participants’ demographic information such as age, travel companions, gender, frequency of rural tourism, occupation, number of companions, frequency of visit Inner Mongolia, type of rural tourism, tourist season, education level, marital status, income, length of trip, tourist purpose, and transportation choices. 

This research used five items to measure place attachment, weather and climate, minority culture, infrastructure, and local culture, respectively [8,9,38,57]. The items for the natural environment were adopted from Kozak and Rimmington [58]. Tourist satisfaction items were used by Oliver [59], and items for intention to revisit Inner Mongolia were extracted from Fishbein and Ajzen [56]. 

### 3.2. Questionnaire Development and Data Collection 

The draft survey was pre-tested by tourism experts to improve the accuracy and confirm the final version of the questionnaire. According to the experts’ feedback, a minor adjustment was made for some questions. The survey investigates rural tourists who visited rural tourism destinations in Inner Mongolia. For data collection, an online questionnaire was used. The data were collected from 29 June to 21 July in 2020. A total of 337 surveys were collected. Among them, 237 usable responses were used for analysis after removing 57 unusable responses.

### 3.3. Demographic Information 

Table 2 presents a demographic summary of the study. Among the sample of 237 participants, 99 were male, and 138 were female. About 19.4% of respondents’ annual household income was less than 10 million CNY, 42.2% was 10 million CNY to below 15 million CNY, 28.3% was between 20 million to below 25 million CNY, and 7.6% was over 25 million CNY. Regarding the respondents’ age, about 8% were less than 25 years old, 49.8 % were between 26 and 30 years old, 36.3% were 31–40 years old, and 5.9% were over 41 years old. The travel partner choice was alone (5.1%), friends (40.1%), family/relatives (48.9%), tour group (3%), and other (3%). In the last three years, the times of traveled rural tourism destination was 3–4 times (38.8%), 5–6 times (34.2%), 1–2 times (21.9%), and over 7 times (5.1%). The most common occupation was teacher (19%), followed by a company employee (16.2%), other (14.8%), tour guide (16%), student (11%), housewife (12.2%), and civil servant (10.1%). The number of travel companions was under 3 people (47.3%), 4–6 people (41.8%), and over 7 people (11%). The number of times to have visited Inner Mongolia was 1–2 times (52.3%), 3–4 times (36.6%), and over 5 times (11.8%). The respondents experienced the type of rural tourism as ethnic tourism (21.5%), eco-tourism (20.4%), village tourism (17.3%), resort tourism (15.6%), agricultural tourism (13.2%), and red tourism (12.1%). The season of tourism visited Inner Mongolia is autumn (37.1%), summer (25.7%), spring (33.8%), and winter (3.4%). Most of the respondents’ degrees were undergraduate (56.1%), followed by graduate degree (20.7%), high school or below (20.3%), and other professional degrees (3%). The length of trip order was 4–6 days (48.1%), under 3 days (24.6%), 7–9 days (22.4%), and over 10 days (4.2%). The purpose of tourist visit to Inner Mongolia is a holiday (70.8%), visiting friends or relatives (11.2%), honeymoon and business (14.4%), and other (3.6%). The most common means of transportation is own vehicle (40.2%), tourist bus (27.4%), train (13.9%), airplane (10.5%), public transport (2.3%), rental vehicle (5.3%), and other (0.4%). 

## 4. Results

### 4.1. Confirmatory Factor Analysis and Data Quality Testing

This research implemented the confirmatory factor analysis to develop the measurement model. The generated model had the acceptable level of goodness-of-fit statistics (χ^2^ = 408.334, df = 271, χ^2^/df = 1.507, *p* < 0.01, RMSEM = 0.046, CFI = 0.954, IFI = 0.895, TLI = 0.944). All measurement items for constructs were significantly loaded. Composite reliability (CR) and Cronbach’s alpha was evaluated to measurement items. As shown in Table 3, the values (place attachment = 0.871, weather and climate = 0.771, minority culture = 0.750, infrastructure = 0.837, Local events = 0.872, natural environment = 0.804, tourist satisfaction = 0.836, intention to revisit Inner Mongolia = 0.855) range was between 0.750 and 0.874, which exceeded the minimum level expected of 0.70 [61]. This result demonstrated the internal consistency among items within a latent factor. 

Average variance extracted (AVE) values were then calculated. As depicted in Table 1, the values (place attachment = 0.635, weather and climate = 0.531, minority culture = 0.501 infrastructure = 0.561, Local events = 0.630, natural environment = 0.672, tourist satisfaction = 0.629, intention to revisit Inner Mongolia = 0.664) were all higher than the minimum threshold of 0.50, as suggested by [61]. This result demonstrated the convergent validity of the multi-item measures. Additionally, the AVE values were all above the correlations between the research variable. In other words, the validity of this research was supported.

### 4.2. Structural Model and Hyphothese Testing

The structural equation model testing results were used to evaluate destination attributes’ effect on tourist satisfaction and intention to revisit Inner Mongolia. The basic criteria of the structural model assessment are coefficient of determination (R^2^) and path coefficient [62]. This research found R^2^ of the endogenous latent constructs for tourist satisfaction (0.572) and intention to revisit Inner Mongolia (0.653). The *β* scores and the corresponding *t* values of the hypothesized relationships among the latent constructs are shown in Table 4. The results demonstrate that place attachment has a significant positive influence on tourist satisfaction (*β* = 0.244, *t* = 2.882), and place attachment directly influences revisit intention (*β* = 0.370, *t* = 4.566). Weather and climate do not have a significant impact on tourist satisfaction (*β* = 0.129, *t* = 1.647), but they have a direct positive and considerable influence on revisit intention (*β* = 0.343, *t* = 4.441). Minority culture has an immediate positive and significant effect on tourist satisfaction (*β* = 0.349, *t* = 3.367) and do not significantly influence revisit intention (*β* = 0.052, *t* = 0.569). Infrastructure directly influences tourist satisfaction (*β* = 0.215, *t* = 2.704) and a significant influence on revisit intention (*β* = 0.200, *t* = 2.709). Local events did not significantly influence tourist satisfaction (*β* = 0.084, *t* = 1.425), and it does not significantly influence revisit intention (*β* = −0.020, *t* = −0.365). The natural environment has no positive and significant influence on tourist satisfaction (*β* = −0.053, *t* = −0.835), but it has a significant positive influence on revisit intention (*β* = 0.155, *t* = 2.568). 

### 4.3. Gender and Tourist Satisfaction and Intention to Revisit Inner Mongolia

**Figure 3:**
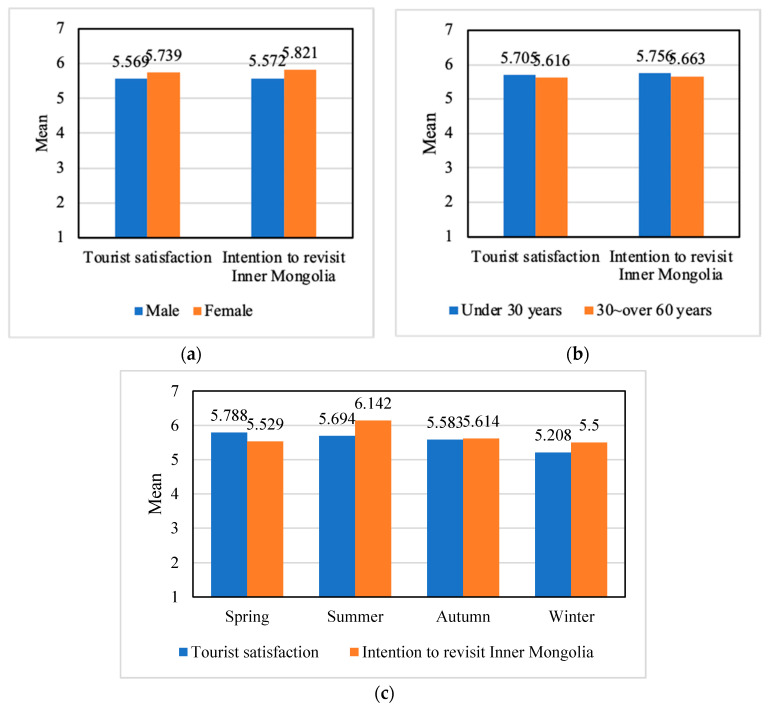
Result of means comparison of model outcomes by demographics of domestic tourists. (**a**) Means comparison of gender; (**b**) means comparison of age; (**c**) means comparison of season.

The findings showed a gender difference in tourism satisfaction: female groups were more satisfied than male groups. On the other hand, the gender groups were no different in terms of revisit intention to Inner Mongolia. In detail, the independent sample t-tests of respondents revealed significant differences in tourist satisfaction and intention to revisit Inner Mongolia across two gender groups (tourist satisfaction: *F* = 4.134, *p* = 0.043; intention to recommend: *F* = 0.289, *p* = 0.592). As shown in Table 5 and Figure 3a, mean score results indicate that scores of tourist satisfaction and intention to revisit Inner Mongolia for the female group were higher than the male group (tourist satisfaction: *M_female_* = 5.569, *M_male_* = 5.739; intention to revisit Inner Mongolia: *M_female_* = 5.572, *M_male_* = 5.821). These findings identified that the female group had a higher desired to visit Inner Mongolia than the male group.

### 4.4. Age and Tourist Satisfaction and Intention to Revisit Inner Mongolia

The findings of the t-test showed that tourist satisfaction and intention to revisit Inner Mongolia did not vary across tourists with different age levels (tourist satisfaction: *F* = 0.106, *p* = 0.745; intention to revisit: *F* = 1.303, *p* = 0.255) (Table 6 and Figure 3b).

### 4.5. Season and Tourist Satisfaction and Intention to Revisit Inner Mongolia

This research also examined season differences in tourist visits in Inner Mongolia (Table 7). The result of one-way analysis of variance (ANOVA) tests revealed significant differences in intention to revisit Inner Mongolia across the season groups, but there was no difference across the season groups for tourism satisfaction (tourist satisfaction: *F* =1.081, *p* = 0.358; intention to revisit Inner Mongolia: *F* = 5.293, *p* = 0.002). As shown in Table 7 and Figure 3c, Mean scores for result indicate scores of tourist satisfaction and intention to revisit Inner Mongolia for season groups. For tourist satisfaction (ranked high to low: *M_spring_* = 5.788, *M_summer_* = 5.694, *M_autumn_* = 5.583, *M_winter_* = 5.208) and intention to revisit Inner Mongolia (ranked high to low: *M_summer_* = 6.142, *M_spring_* = 5.529, *M_autumn_* = 5.614, *M_winter_* = 5.500). In addition, a post hoc analysis was conducted using Fisher’s LSD. The results indicated that the tourist satisfaction did not significantly differ between any pair of season groups. On the contrary, Fisher’s LSD analysis showed a significant difference between intention to revisit Inner Mongolia and season groups (Figure 3).

## 5. Discussion and Implication

Even though there are many rural tourism destinations in China, many other destinations are still unknown to the public, like Inner Mongolia. This study investigated the relationships among Inner Mongolia destination attributes, tourist satisfaction, and revisit intention from the domestic tourist perspectives. This research utilized a survey method and quantitative approach. The findings suggest that tourist satisfaction and destination attributes of place attachment, minority culture, and infrastructure have a positive relationship. This study also shows that destination attributes of place attachment, weather and climate, infrastructure, and natural environment have a positive relationship with revisit intention. As the previous studies indicated [9,63], destination attributes positively affect satisfaction and behavior intention.

Moreover, the proposed theoretical framework satisfactorily accounted for the total variance of tourist satisfaction and revisit intention. This study provides a new perspective by investigating the demographic characteristics of tourist satisfaction and revisit intention. The findings of gender difference in this study are in accordance with previous studies’ results, which show the significant variation of satisfaction between female and male tourists [64,65]. The results can help destination marketers in Inner Mongolia to acknowledge the characteristics of their target segment. From a new perspective in this research, tourists are considered a positive influence variable demographic characteristic over four seasons and revisit intention. The tourists have more intention to revisit Inner Mongolia in summer than other seasons. These findings have important implications when destination marketing facilitates the achievement of local development. 

Previous studies have documented the destination attributes’ positive effectiveness in improving tourist satisfaction and revisit intention [9,12,42,66,67]. The structural equation model results showed that the place attachment, minority culture, and infrastructure positively correlate with tourist satisfaction. These results align with previous research findings [8,68,69]. One unanticipated finding was that, unlike previous studies [9,70], the infrastructure has a positive relationship with tourist satisfaction. Our research also suggested that destination attributes (place attachment, weather and climate, infrastructure, and natural environment) positively affect tourist revisit intention. Therefore, destination managers, tourism enterprises, and local administrations need to develop local cultural activities or events that help to attract more tourists. For example, equestrian/equine tourism is a growing niche market in rural areas, and horse riding has been incorporated into various commercial tourism products worldwide [14]. Furthermore, horses have played an inseparable role in the history of Mongolians and their culture. Tourists can experience riding horses or watch competitions.

Previous research showed that tourist behaviors tend to differ based on tourist characteristics [52,64]. This research revealed that, except for gender, there were no differences in tourist satisfaction. According to the mean comparison results, female tourists are more satisfied than male tourists. Therefore, destination managers, tour enterprises, and local administrations can target the female tourist as the priority in target marketing of Inner Mongolia rural tourism. Admittedly, male tourists, regarding their satisfaction and revisit intention, may vary depending on the type of tourism products and experience. This study assumes that male tourists might be more satisfied and intend to revisit Inner Mongolia when asked to rate their satisfaction and revisit intention regarding adventure travel or experience of Equestrian/equine tourism. Furthermore, one-way ANOVA analysis showed that the observed difference between season and revisit intention was statistically significant. From the aspect of the season, tourists are more likely to revisit Inner Mongolia in summer. On average, the peak season of Inner Mongolia is summer [71,72]. Inner Mongolia belongs to the temperate continental and monsoon climate. For tourists, the most comfortable season to visit Inner Mongolia is summer. To draw more attention, decision-makers and local administrations can develop and promote other season’s tourism products, for example, winter sports activities or the gorgeous charm of the desert in autumn. 

Tourists’ revisit intention of Inner Mongolia in the summer is significantly more than in the winter. This result means it is necessary to improve the destination attribute of Inner Mongolia and develop the tourism products based on different seasons in Inner Mongolia. For example, in spring, the mausoleum of Genghis Khan spring ritual is the grandest at the shrine. Autumn is the best time to visit the *Populus euphratica* forest in Ejina. In winter, an excellent ice and snow resource is found in the Arxan-Chaihe location, and it has a natural advantage for winter activities. In this vein, destination managers and local administrations need to promote compelling and unique minority destination resources to give destinations a competitive advantage.

A noteworthy finding in this study is the demography of respondents. The most portion of respondents’ occupations is teachers (19%), the purpose of visit Inner Mongolia is holiday (70.8%), and 40.2% of those who visit Inner Mongolia use their vehicle. Moreover, 30% of respondents were from North China. In China, teachers usually have a long vacation in summer and winter. This finding is a significant opportunity to extend the segment of tourist groups. Self-driving trips have grown in popularity in China. In 2018, 580 million domestic tourists traveled by their own vehicle [73]. These findings were an unconventional discovery from the previous research.

Finally, several significant limitations need to be considered in this research. First, this study’s target population was domestic tourists, so it was challenging to represent international tourists’ views of the Inner Mongolia destination. Future researchers are recommended to increase the broader range of participants to include international visitors. Second, another limitation is that the current study explored few dimensions of destination attributes. Future studies can conduct qualitative research or extend the dimensions of factors to explore possible different destination attributes that might influence tourist behavior. Finally, the data were collected by internet survey method. Thus, participants were restricted by computer or cellphone users. Therefore, future research should examine tourist behavior in a local setting to overcome this problem and improve validity.

## 6. Conclusions

After the COVID-19 outbreak, China has implemented strict containment measures to control the spread of the virus. As a result, Chinese domestic tourists have a strong desire to go outside and travel. In particular, rural tourism has become the first choice for Chinese tourists to travel for leisure to fulfill their travel desire [13]. The unique destination physiography and climate are competitive advantages of determinant attributes [8,18]. As the result of the current study indicated, weather and climate positively impact tourist satisfaction. This means that tourists prefer to visit Inner Mongolia in the most comfortable season, namely summer. Tourism enterprises therefore should develop appropriate tourism products and create a summer holiday tourism destination. The contrast between urban life and rural tourism choice would be a determinant in redirecting the concept of an attractive tourism destination, but traveling to remote places could offer memorable tourism experiences [8,10]. Khadaroo and Seetanah [74] argue that the infrastructure is an essential demand for international tourists. In particular, European and American tourists are sensitive to transport and non-transport infrastructure [74]. As a huge potential tourism destination, Inner Mongolia should cooperate with the government to improve tourism infrastructure in the destination to attract more visitors. Finally, to enhance tourist demands, the tourism enterprises, industrial practitioners, and local administrations should strengthen publicity and encourage enterprises to promote the Inner Mongolia tourism industry to attract more tourists, especially international tourists. We hope that this research contributes to the Inner Mongolia tourism development and tourism activities.

## Figures and Tables

**Table 1:** Tourist reception and Income of tourism in Inner Mongolia.

	Number of Tourist Reception			Income of Tourism		
Year	Domestic Tourism	International Tourist	Total	Domestic Tourism	International Tourism	Total
2010	4478	142.8	4620.8	692.9	6	732.7
2011	5178	151.5	5329.5	847.3	6.7	889.6
2012	5887	159.2	6046.2	1080.7	7.7	1128.5
2013	6613	161.6	6774.6	1343.7	9.6	1403.5
2014	7414.9	167.1	7585	1745	10	1805.3
2015	8351.8	160.8	8512.6	2193.8	9.6	2257.1
2016	9627.4	177.9	9805.5	2635.6	11.4	2714.7
2017	11,461.2	184.8	11,646	3358.6	12.5	3440.1
2018	12,856.1	188.1	13,044.2	3924	12.7	4011.4
2019	19,316.7	195.8	19,512.5	4558.5	13.4	4651.5
2020 *	8192.4	8.68	8200.98	1462	0.34	1464.38

Note. * The data for 2020 are from January to September. Note. The number of tourist reception: 10,000 person-times; Income of domestic tourism: 100 million CNY; Income of international tourist: 10,000 USD; Source: The Inner Mongolia Autonomous Region Culture and Tourism Department, Inner Mongolia Statistical Yearbook.

**Table 2:** Description of the respondents (*n* = 237).

Variables	Frequency	Percent (%)	Variables	Frequency	Percent (%)
Travel partner			During which season did you visit Inner Mongolia?		
Alone	12	5.1	Spring	80	33.8
Friends	95	40.1	Summer	61	25.7
Family/relatives	116	48.9	Autumn	88	37.1
Tour group	7	3.0	Winter	8	3.4
Other	7	3.0			
Times of visit rural tourism in the last three years			Education		
1–2 times	52	21.9	High school degree or below	48	20.3
3–4 times	92	38.8	Undergraduate degree	133	56.1
5–6 times	81	34.2	Graduate degree	49	20.7
Over 7 times	12	5.1	Other professional degree	7	3.0
Age			Gender		
Under 18	1	0.4	Male	114	40.7
18~25	18	7.6	Female	166	59.3
26~30	118	49.8	Marital status		
31~40	86	36.3	Single	135	57.0
41~50	11	4.6	Married	101	42.6
51~60	3	1.3	Other	1	0.4
Occupation			Household income in US Dollars (yearly):		
Student	26	11.0	less than 10 million CNY	46	19.4
Company employee	40	16.2	10-below15 million CNY	100	42.2
Civil servant	24	10.1	15-below20 million CNY	67	28.3
Teacher	45	19.0	20-below25 million CNY	6	2.5
Housewife	29	12.2	25-below30 million CNY	6	2.5
Tour guide	38	16.0	Over 30 million CNY	12	5.1
other	35	14.8			
How many people travelled with you?			Length of trip		
Under 3 person	112	47.3	Under 3 days	60	25.3
4–6 person	99	41.8	4–6 days	114	48.1
7–9 person	7	3.0	7–9 days	53	22.4
Over 10 person	19	8.0	Over 10 days	10	4.2
Times of visited Inner Mongolia			Purpose of visit to Inner Mongolia?		
1–2 times	124	52.3	Holiday	177	70.8
3–4 times	64	36.6	Honeymoon	20	8.0
5–6 times	2	0.8	Business	16	6.4
Over 7 times	26	11.0	Visiting friends or relatives	28	11.2
			Other	9	3.6
Which type of rural tourism did you visit?			Transportation		
Resort tour	85	15.6	Airplane	28	10.5
Agricultural tour	71	13.2	Own vehicle	107	40.2
Village tour	93	17.3	Tourist bus	73	27.4
Eco tour	110	20.4	Rental vehicle	14	5.3
Ethnic tour	116	21.5	Train	37	13.9
Red tour	65	12.1	Public transport	6	2.3
			Other	1	0.4

**Table 3:** Summary of the confirmatory factor analysis result.

Variable	(1)	(2)	(3)	(4)	(5)	(6)	(7)	(8)	CR	AVE
(1) Tourist satisfaction	0.793	0.376 ^b^	0.206	0.448	0.319	0.023	0.012	0.329	0.836	0.629
(2) Place attributes	0.613 ^a^	0.797	0.197	0.370	0.242	0.007	0.038	0.461	0.874	0.635
(3) Weather and climate	0.454	0.444	0.729	0.212	0.086	0.033	0.088	0.397	0.771	0.531
(4) Minority culture	0.669	0.608	0.460	0.707	0.303	0.002	0.026	0.316	0.750	0.501
(5) Infrastructure	0.565	0.492	0.293	0.550	0.749	0.004	0.004	0.266	0.837	0.561
(6) Local event	0.152	0.083	0.181	0.045	0.067	0.794	0.013	0.011	0.872	0.630
(7) Natural environment	0.109	0.196	0.296	0.160	0.064	0.114	0.820	0.122	0.804	0.672
(8) Intention to revisit Inner Mongolia	0.574	0.679	0.630	0.562	0.516	0.106	0.349	0.815	0.855	0.664

Note: Model measurement fit: x^2^ = 408.334 (df = 271, *p* < 0.001), x^2^/df = 1.507, RMSEA = 0.046, CFI = 0.954, NFI = 0.875, RFI = 0.851, IFI = 0.954, TLI = 0.944. ^a^ Correlation between variables. ^b^ Squared correlation.

**Table 4:** Determination of destination attributes.

Destination Attributes	Tourist Satisfaction (Sat)		Intention to Revisit Inner Mongolia (RI)	
	Beta	*t*-Value	Beta	*t*-Value
Place attachment (PA)	0.244	2.882 **	0.370	4.566 *
Weather and climate (WC)	0.129	1.647	0.343	4.441 **
Minority culture (MC)	0.349	3.367 **	0.052	0.569
Infrastructure (I)	0.215	2.704 **	0.200	2.709 *
Local events (LE)	0.084	1.425	−0.020	−0.365
Natural environment (Env)	−0.053	−0.835	0.155	2.568 *

R^2^ (Adjusted), Tourist satisfaction = 0.572, Intention to revisit Inner Mongolia = 0.653. Model measurement fit: x^2^ = 409.005 (df = 272, *p* < 0.01), x^2^/df = 1.504, RMSEA = 0.046, CFI = 0.954, NFI = 0.875, RFI = 0.851, IFI = 0.954, TLI = 0.945. * *p* < 0.05 and ** *p* < 0.01.

**Table 5:** Gender differences in tourist satisfaction and Intention to revisit Inner Mongolia.

Variables	Gender	N	Mean	SD	*F*-Value	*p*-Value
Tourist satisfaction	Male	99	5.569	1.176	4.134	0.043
	Female	138	5.739	0.927		
Intention to revisit Inner Mongolia	Male	99	5.572	1.038	0.289	0.592
	Female	138	5.821	0.967		

**Table 6:** Age differences in tourist satisfaction and intention to revisit Inner Mongolia.

Variables	Age	N	Mean	SD	*F*-Value	*p*-Value
Tourist satisfaction	Under 30 years	137	5.705	1.018	0.106	0.745
	30~over 60 years	100	5.616	1.072		
Intention to revisit Inner Mongolia	Under 30 years	137	5.756	1.014	1.303	0.255
	30~over 60 years	100	5.663	0.991		

**Table 7:** Results of ANOVA: season differences in tourist satisfaction and Intention to revisit Inner Mongolia.

Variables	Season	N	Mean	SD	*F*-Value	*p*-Value
Tourist satisfaction	Spring	80	5.788	0.979	1.081	0.358
	Summer	61	5.694	1.184		
	Autumn	88	5.583	0.916		
	Winter	8	5.208	1.642		
Intention to revisit Inner Mongolia	Spring	80	5.529	0.796	5.293	0.002
	Summer	61	6.142	1.195		
	Autumn	88	5.614	0.933		
	Winter	8	5.500	1.003		

## Data Availability

The dataset used in this research are available upon request from the corresponding author. The data are not publicly available due to restrictions.

## References

[1] Liu C., Dou X., Li J., Cai L.A. (2020). Analyzing government role in rural tourism development: An empirical investigation from China. J. Rural. Stud..

[2] Lu S., Li G., Xu M. (2020). The linguistic landscape in rural destinations: A case study of Hongcun Village in China. Tour. Manag..

[3] Su B. (2011). Rural tourism in China. Tour. Manag..

[4] Su B. (2013). Developing Rural Tourism: The PAT Program and ‘Nong jia le’ Tourism in China. Int. J. Tour. Res..

[5] The State Council of the People’s Republic of China The First List of Rural Tourism Key Villages in China. http://www.gov.cn/fuwu/2019-07/30/content_5416558.htm.

[6] The State Council of the People’s Republic of China The Second List of Rural Tourism Key Villages in China. http://www.gov.cn/zhengce/zhengceku/2020-09/04/content_5540367.htm.

[7] Gao J., Wu B. (2017). Revitalizing traditional villages through rural tourism: A case study of Yuanjia Village, Shaanxi Province, China. Tour. Manag..

[8] Kim J.H. (2014). The antecedents of memorable tourism experiences: The development of a scale to measure the destination attributes associated with memorable experiences. Tour. Manag..

[9] Moon H., Han H. (2018). Destination attributes influencing Chinese travelers’ perceptions of experience quality and intentions for island tourism: A case of Jeju Island. Tour. Manag. Perspect..

[10] Rainero C., Modarelli G. (2020). The attractive power of rural destinations and a synergistic community cooperative approach: A “tourismability” case. Sustainability.

[11] Roman M., Roman M., Niedziółka A. (2020). Spatial Diversity of Tourism in the Countries of the European Union. Sustainability.

[12] Eom T., Han H., Song H. (2020). Discovering the perceived attributes of CBT destination travelers in South Korea: A mixed method approach. Tour. Manag..

[13] Zhu H., Deng F. (2020). How to Influence Rural Tourism Intention by Risk Knowledge during COVID-19 Containment in China: Mediating Role of Risk Perception and Attitude. Int. J. Environ. Res. Public Health.

[14] Lane B., Kastenholz E. (2015). Rural tourism: The evolution of practice and research approaches–towards a new generation concept?. J. Sustain. Tour..

[15] Kastenholz E., Marques C.P., Carneiro M.J. (2020). Place attachment through sensory-rich, emotion-generating place experiences in rural tourism. J. Dest. Mark. Manag..

[16] Buhalis D. (2000). Marketing the competitive destination of the future. Tour. Manag..

[17] Lew A.A. (1987). A framework of tourist attraction research. Ann. Tour. Res..

[18] Crouch G.I., Ritchie J.B. (1999). Tourism, competitiveness, and societal prosperity. J. Bus. Res..

[19] Schlesinger W., Cervera-Taulet A., Pérez-Cabañero C. (2020). Exploring the links between destination attributes, quality of service experience and loyalty in emerging Mediterranean destinations. Tour. Manag. Perspect..

[20] Ekanayake I.E., Gnanapala A.C. (2016). Travel experiences and behavioural intentions of the tourists: A study on eastern province of Sri Lanka. Tour. Leis. Glob. Chang..

[21] Sangpikul A. (2018). The effects of travel experience dimensions on tourist satisfaction and destination loyalty: The case of an island destination. Int. J. Cult. Tour. Hosp. Res..

[22] Smith S.L. (1994). The tourism product. Ann. Tour. Res..

[23] Turner L., Reisinger Y. (1999). Importance and expectations of destination attributes for Japanese tourists to Hawaii and the gold coast compared. Asia Pac. J. Tour. Res..

[24] Şahin İ., Güzel F.Ö. (2020). Do experiential destination attributes create emotional arousal and memory?: A comparative research approach. J. Hosp. Mark. Manag..

[25] Eusébio C., Vieira A.L. (2013). Destination attributes’ evaluation, satisfaction and behavioural intentions: A structural modelling approach. Int. J. Tour. Res..

[26] Ozturk U.A., Gogtas H. (2016). Destination attributes, satisfaction, and the cruise visitor’s intent to revisit and recommend. Tour. Geogr..

[27] Som A.P.M., Marzuki A., Yousefi M. (2012). Factors influencing visitors’ revisit behavioral intentions: A case study of Sabah, Malaysia. Int. J. Mark. Stud..

[28] Oliver R.L. (1980). A cognitive model of the antecedents and consequences of satisfaction decisions. J. Mark. Res..

[29] Jani D., Han H. (2014). Personality, satisfaction, image, ambience, and loyalty: Testing their relationships in the hotel industry. Int. J. Hosp. Manag..

[30] Simcock P., Sudbury L., Wright G. (2006). Age, perceived risk and satisfaction in consumer decision making: A review and extension. J. Mark. Manag..

[31] Tuu H.H., Olsen S.O. (2009). Food risk and knowledge in the satisfaction-repurchase loyalty relationship. Asia Pacific J. Mark. Logist..

[32] Kozak M. (2003). Measuring tourist satisfaction with multiple destination attributes. Tour. Anal..

[33] Cervi L. (2019). Travellers’ virtual communities: A success story. Univ. Rev. Cienc. Soc. Hum..

[34] Bernini C., Cagnone S. (2014). Analysing tourist satisfaction at a mature and multi-product destination. Curr. Issues Tour..

[35] Kozak M., Rimmington M. (2000). Tourist satisfaction with Mallorca, Spain, as an off-season holiday destination. J. Travel Res..

[36] Tribe J., Snaith T. (1998). From SERVQUAL to HOLSAT: Holiday satisfaction in Varadero, Cuba. Tour. Manag..

[37] Ritchie J.B., Crouch G.I. (2003). The Competitive Destination: A Sustainable Tourism Perspective.

[38] Crouch G.I. (2011). Destination competitiveness: An analysis of determinant attributes. J. Travel Res..

[39] Cole S.T., Scott D. (2004). Examining the mediating role of experience quality in a model of tourism experiences. J. Travel. Tour. Mark..

[40] Kozak M. (2001). Repeaters’ behavior at two distinct destinations. Ann. Tour. Res..

[41] Um S., Chon K., Ro Y. (2006). Antecedents of revisit intention. Ann. Tour. Res..

[42] Meng B., Han H. (2018). Working-holiday tourism attributes and satisfaction in forming word-of-mouth and revisit intentions: Impact of quantity and quality of intergroup contact. J. Dest. Mark. Manag..

[43] Sinclair-Maragh G. (2017). Demographic analysis of residents’ support for tourism development in Jamaica. J. Dest. Mark. Manag..

[44] Tsui A.S., O’reilly III C.A. (1989). Beyond simple demographic effects: The importance of relational demography in superior-subordinate dyads. Acad. Manag. J..

[45] Pfeffer J. (1985). Organizational demography: Implications for management. Calif. Manag. Rev..

[46] Huh J., Uysal M., McCleary K. (2006). Cultural/heritage destinations: Tourist satisfaction and market segmentation. J. Hospit. Leis. Market..

[47] Kozak M., Bigné E., Andreu L. (2005). Satisfaction and destination loyalty: A comparison between non-repeat and repeat tourists. J. Qual. Assur. Hosp. Tour..

[48] Tsiotsou R., Vasioti E. (2006). Using demographics and leisure activities to predict satisfaction with tourism services in Greece. J. Hospit. Leisure. Market..

[49] Ozdemir B., Aksu A., Ehtiyar R., Çizel B., Çizel R.B., İçigen E.T. (2012). Relationships among tourist profile, satisfaction and destination loyalty: Examining empirical evidences in Antalya region of Turkey. J. Hosp. Mark. Manag..

[50] Wang C., Qu H., Hsu M.K. (2016). Toward an integrated model of tourist expectation formation and gender difference. Tour. Manag..

[51] Pung J.M., Yung R., Khoo-Lattimore C., Del Chiappa G. (2020). Transformative travel experiences and gender: A double duoethnography approach. Curr. Issues. Tour..

[52] Han H., Hsu L.T.J., Lee J.S., Sheu C. (2011). Are lodging customers ready to go green? An examination of attitudes, demographics, and eco-friendly intentions. Int. J. Hosp. Manag..

[53] Han H., Hsu L.T.J., Lee J.S. (2009). Empirical investigation of the roles of attitudes toward green behaviors, overall image, gender, and age in hotel customers’ eco-friendly decision-making process. Int. J. Hosp. Manag..

[54] Homburg C., Giering A. (2001). Personal characteristics as moderators of the relationship between customer satisfaction and loyalty—an empirical analysis. Psychol. Mark..

[55] Im S., Bayus B.L., Mason C.H. (2003). An empirical study of innate consumer innovativeness, personal characteristics, and new-product adoption behavior. J. Acad. Mark. Sci..

[56] Fishbein M., Ajzen I. (1975). Belief. Attitude, Intention and Behavior: An Introduction to Theory and Research.

[57] Hidalgo M.C., Hernandez B. (2001). Place attachment: Conceptual and empirical questions. J. Environ. Psychol..

[58] Kozak M., Rimmington M. (1999). Measuring tourist destination competitiveness: Conceptual considerations and empirical findings. Int. J. Hosp. Manag..

[59] Oliver R.L. (2010). Customer Satisfaction.

[60] Saleh F., Ryan C. (1991). Analysing service quality in the hospitality industry using the SERVQUAL model. Serv. Ind. J..

[61] Hair J.F., Black W.C., Babin B.J., Anderson R.E. (2010). Multivariate Data Analysis.

[62] Hair J.F., Ringle C.M., Sarstedt M. (2013). Partial least squares structural equation modeling: Rigorous applications, better results and higher acceptance. Long. Range. Plan..

[63] Han H., Al-Ansi A., Olya H.G., Kim W. (2019). Exploring halal-friendly destination attributes in South Korea: Perceptions and behaviors of Muslim travelers toward a non-Muslim destination. Tour. Manag..

[64] Al-Ansi A., Olya H.G., Han H. (2019). Effect of general risk on trust, satisfaction, and recommendation intention for halal food. Int. J. Hosp. Manag..

[65] Hwang J., Han H., Kim S. (2015). How can employees engage customers?. Int. J. Contemp. Hosp. Manag..

[66] Hwang S.N., Lee C., Chen H.J. (2005). The relationship among tourists’ involvement, place attachment and interpretation satisfaction in Taiwan’s national parks. Tour. Manag..

[67] Yang J., Ryan C., Zhang L. (2013). Ethnic minority tourism in China–Han perspectives of Tuva figures in a landscape. Tour. Manag..

[68] Ramkissoon H., Smith L.D.G., Weiler B. (2013). Testing the dimensionality of place attachment and its relationships with place satisfaction and pro-environmental behaviours: A structural equation modelling approach. Tour. Manag..

[69] Yang L. (2012). Tourists’ perceptions of ethnic tourism in Lugu Lake, Yunnan, China. J. Herit. Tour..

[70] Albayrak T., Caber M. (2016). Destination attribute effects on rock climbing tourist satisfaction: An Asymmetric Impact-Performance Analysis. Tour. Geogr..

[71] Ge Q., Yang X., Qiao Z., Liu H., Liu J. (2014). Monitoring Grassland Tourist Season of Inner Mongolia, China Using Remote Sensing Data. Adv. Meteorol..

[72] Buckley R., Ollenburg C., Zhong L. (2008). Cultural landscape in Mongolian tourism. Ann. Tour. Res..

[73] China Daily Website Self-driving Trips Popular in Holiday as Chinese Embrace Outdoors. http://www.chinadaily.com.cn/a/202005/08/WS5eb4b294a310a8b2411541b2.html.

[74] Khadaroo J., Seetanah B. (2007). Transport infrastructure and tourism development. Ann. Tour. Res..

